# Glia and TRPM2 Channels in Plasticity of Central Nervous System and Alzheimer's Diseases

**DOI:** 10.1155/2016/1680905

**Published:** 2016-01-28

**Authors:** Jing Wang, Michael F. Jackson, Yu-Feng Xie

**Affiliations:** ^1^Key Laboratory of Orthopedics of Gansu Province, The Second Hospital of Lanzhou University, No. 82 Cui Ying Men, Lanzhou, Gansu 730030, China; ^2^Department of Pharmacology & Therapeutics, University of Manitoba, Canada; ^3^Kleysen Institute for Advanced Medicine, University of Manitoba, 710 William Avenue, SR426 Winnipeg, MB, Canada R3E 0Z3; ^4^Leslie Dan Faculty of Pharmacy, University of Toronto, 144 College Street, Toronto, ON, Canada M5S 3M2

## Abstract

Synaptic plasticity refers to the ability of neurons to strengthen or weaken synaptic efficacy in response to activity and is the basis for learning and memory. Glial cells communicate with neurons and in this way contribute in part to plasticity in the CNS and to the pathology of Alzheimer's disease (AD), a neurodegenerative disease in which impaired synaptic plasticity is causally implicated. The transient receptor potential melastatin member 2 (TRPM2) channel is a nonselective Ca^2+^-permeable channel expressed in both glial cells (microglia and astrocytes) and neurons. Recent studies indicated that TRPM2 regulates synaptic plasticity as well as the activation of glial cells. TRPM2 also modulates oxidative stress and inflammation through interaction with glial cells. As both oxidative stress and inflammation have been implicated in AD pathology, this suggests a possible contribution of TRPM2 to disease processes. Through modulating the homeostasis of glutathione, TRPM2 is involved in the process of aging which is a risk factor of AD. These results potentially point TRPM2 channel to be involved in AD through glial cells. This review summarizes recent advances in studying the contribution of TRPM2 in health and in AD pathology, with a focus on contributions via glia cells.

## 1. Introduction

Inflammation, oxidative stress, and disturbance of intracellular Ca^2+^ ([Ca^2+^]_i_) homeostasis are the most common signaling pathways contributing to many neuropathological conditions and/or diseases, such as Alzheimer's disease (AD), prion-related diseases, parkinsonism-dementia, and chronic neuropathic/inflammatory pain [1–4]. These neuropathological changes are associated with not only the roles played by neurons but also the activation of glial cells (mainly including microglia and astrocytes) and the interaction between neurons and glial cells [1, 2]. Central sensitization is an enhanced state of excitatory synaptic transmission in nociceptive neurons and is a specific form of synaptic plasticity involving neurons and glial cells in the central nervous system (CNS) [5–8]. Synaptic plasticity is the ability of neurons to change the transmission efficacy at synapses to adapt to different conditions, involves glial cells, and is thought of as the mechanism of learning and memory [2, 9–12]. Alzheimer's disease (AD) is a neurodegenerative disease characterized by progressive decline of recognition with advanced age and involves the pathophysiological changes of N-methyl-D-aspartate (NMDA) receptor which is also involved in central sensitization and synaptic plasticity [13–15]. Therefore, through inflammation, glutamate receptor involvement, and neuron-glia communication [2, 6, 7, 16–18], both the central sensitization and synaptic plasticity may be involved in the pathology AD.

As a newly identified nonselective Ca^2+^-permeable cation channel and the sensor of reactive oxygen species (ROS), transient receptor potential melastatin member 2 (TRPM2) channel has recently been indicated to be involved in inflammatory/neuropathic pain, synaptic plasticity, oxidative stress, and neurodegenerative diseases through modulation of multiple signaling pathways [17–20]. In addition to being expressed in neurons, the TRPM2 channel is also found to be expressed in glial cells (microglia and astrocytes) and plays important role in pathophysiological conditions [21]. Therefore, TRPM2 channel is an important regulator of plasticity, not only in health but also in AD which is characterized by synapse loss and involves inflammation and oxidative stress [1, 13].

## 2. TRPM2 Channel Expression in Glial Cells

The glial cells in the CNS mainly include microglia, astrocytes, and oligodendrocytes. The microglia in the CNS function as quiescent immune cells that maintain the homeostasis of brain through surveying the environment and scavenging debris. The astrocytes regulate multiple aspects of neurons and synaptic functions throughout the lifetime, including synapse formation and uptake and recycling of neurotransmitters. Recent study indicates that glial cells also express the TRPM2 channel which plays an important role in immune and inflammatory responses [22, 23]. The protein and mRNA of TRPM2 channel are both confirmed to be expressed in spinal microglia [17, 21, 24, 25]. Consistently, TRPM2-mediated Ca^2+^ current can be detected in cultured microglia [24, 26] while inhibiting the expression of TRPM2 channel by introduction of small interfering RNA (siRNA) into the astrocytes can reduce the inflammation-induced oxidative stress [21]. These results suggest that TRPM2 channel is expressed in microglia and astrocytes in the CNS at both transcriptional and posttranscriptional levels and functions well in these cells.

In addition, the expression of TRPM2 channel in glial cells is affected by multiple stimulations and plays important role in behavior. For example, the expression of TRPM2 mRNA can be increased by cytokine interleukin-1*β* (IL-1*β*) in human C13 microglial cells [24]. Oxidative stress can enhance the expression of TRPM2 mRNA in astrocytes through influx of extracellular Ca^2+^ [27]. In carrageenan-induced inflammation and sciatic nerve injury, the expression of TRPM2 mRNA in the inflamed paw and areas around the injured sciatic nerve is increased [17]. In addition, the Ca^2+^ signaling induced by lipopolysaccharide and interferon gamma (LPS/IFN*γ*) in microglia is absent by pharmacological blockade or gene deletion of TRPM2 channel [28] while deletion of TRPM2 channel attenuates the activation of spinal microglia in the neuropathic pain model with peripheral nerve injury [22, 23]. These studies imply that glial TRPM2 channel may play an important role in the plasticity of the CNS and neurodegenerative diseases such as AD, since the Ca^2+^ signaling, oxidative stress, and inflammation/nerve injury are involved in the plasticity of the CNS and the pathology of AD.

## 3. Glia and TRPM2 Channel in Central Sensitization and Synaptic Plasticity in CNS

Central sensitization, is a specific use-dependent plasticity of nociceptive neurons in the CNS, can result in pain under normally innocuous stimulus after inflammation or injury, and is thought of as a crucial mechanism underlying the increased excitability of nociceptive pathways in the CNS [5]. Previous studies [6, 7, 29] indicate that inflammatory stimulation of the tooth pulp produces central sensitization of nociceptive neurons in the trigeminal subnucleus caudalis mediated by glutamate, ATP, and mitogen-activated protein kinase p38 (p38MAPK) signaling which are well-known to be involved in the synaptic plasticity [30–32]. In parallel, excitatory synaptic transmission in spinal cord slices, long-term potentiation (LTP, a form of synaptic plasticity to underlie the basic molecular mechanism of learning and memory) in the intact spinal cord, and the central sensitization-driven pain hypersensitivity are impaired in toll-like receptor knock-out mice [33]. Astrocytes can release ATP, causing significant attenuation of synaptic inhibition in the pyramidal neurons and facilitating the induction of LTP through neuron-glia communication and action on cannabinoid receptor [34, 35]. Microglia can prune unnecessary synapses and axon terminals during postnatal development and adaptation to novel environments, which plays important role in synaptic remodeling [36, 37]. These studies imply that both central sensitization and synaptic plasticity are involved in learning and memory through the activities of glial cells. This hypothesis is further supported by the study finding that both anxiety and chronic pain are capable of blocking the presynaptic LTP [38].

The involvement of glial cells in sensitization and plasticity is suggested to be related with the TRPM2 channel expressed in glial cells. The TRPM2 channel in microglia and astrocyte is found to be involved in the neurotoxicity mediated by p38MAPK, c-Jun N-terminal kinase (c-JNK), and nuclear factor kappa-B (NF*κ*B) signaling [21] while p38MAPK is involved in the central sensitization mediated by glial cells [7] and in the synaptic plasticity [30]. The Ca^2+^ signaling induced by inflammatory molecules, LPS/IFN*γ*, in microglia from wild-type mice is absent after pharmacological blockade or gene deletion of TRPM2 channel, while the Ca^2+^ signaling is a mechanism for activation of microglia [28]. Furthermore, the p38MAPK and JNK signaling is suggested to contribute to the LPS/IFN*γ*-induced activation of microglia mediated by TRPM2 channel [28]. In the neuropathic pain models induced by peripheral nerve injury, the deletion of TRPM2 channel attenuates the neutrophil infiltration through the activation of spinal microglia and the production of chemokine ligand-2 from macrophages around the damaged peripheral nerve [17, 22, 23]. Furthermore, it is found that TRPM2 knock-out mice demonstrate attenuation of nocifensive behaviors in formalin test, mechanical allodynia, and thermal hyperalgesia in carrageenan-induced inflammatory pain and sciatic nerve injury-induced neuropathic pain models [17]. The activation of microglia by nerve injury and the glial chemokines are suppressed by knock-out of TRPM2 channel [17]. Previous studies indicate that the TRPM2 knock-out mice also demonstrate decreased PSD95 and phosphorylation of glycogen synthase kinase-3*β* (GSK3*β*), impaired long-term depression (LTD, another form of synaptic plasticity) [39]. These results imply that the TRPM2 channel expressed in glial cells is involved in the plasticity of CNS in neuropathic and inflammatory pain through aggravating pronociceptive response, which requires further elucidation using specific deletion of TRPM2 channel in glial cells.

## 4. Glia and TRPM2 Channel in Alzheimer's Diseases

There is increasing evidence suggesting that the pathophysiology of neurodegenerative disorders is related to the inflammatory responses and oxidative stress mediated by microglia through producing neurotoxic factors such as proinflammatory cytokines and nitric oxide that lead to neuronal degeneration [2]. It is found that microglia can be activated by transthyretin amyloid accumulation which in the CNS can cause a kind of fatal and untreatable genetic disease, oculoleptomeningeal amyloidosis, leading to the secretion of inflammatory molecules such as tumor necrosis factor-*α* (TNF-*α*), interleukin-6 (IL-6), and nitric oxide, and the neuronal damage [40]. The release of ATP from cortical astrocytes decreases following the age, which impairs the astrocytic modulation of synaptic transmission in neocortex and therefore contributes to the impairment of synaptic plasticity and the age-related decline of cognition [34, 35]. These studies suggest that the glial cells and the neuron-glia communication in the CNS are involved in the functions of brain and the pathology of AD.

AD, a neurodegenerative disorder exhibiting a gradual decline in cognitive function, is characterized by the presence of neuritic plaques composed of neurofibrillary tangles and amyloid beta (A*β*) peptide. Animals treated with A*β* show impaired ability of learning and memory, activated astrocytes and microglial cells, and disturbed activation of c-JNK and GSK3*β* [41], suggesting activation of glial cells in AD pathology. Astrocytes around the amyloid plaques are found to be activated to produce GABA by monoamine oxidase-B and release GABA through the bestrophin 1 channel. Through acting on presynaptic GABA receptors, the released GABA from astrocytes is capable of decreasing the spike probability of granule cells in the dentate gyrus of AD model mice, impairing the synaptic plasticity and learning and memory [42]. These results provide solid support for the proposal that glial cells are involved in AD. In AD, the neuropathological characteristics of the formation of senile plaques by A*β* is associated with the chronic inflammation involving reactivated astrocytes, microglia, and proinflammatory molecules such as IL-1*β*, TNF-*α*, human CCAAT/enhancer-binding protein (CEBP) delta (CEBPD), p38MAPK, and GSK3*β*. In amyloid precursor protein (APP) transgenic mice, astrocytic CEBPD is associated with the activation and migration of microglia [43]. Furthermore, A*β* derived from transgenic mice is found to be accumulated initially on neurite membranes with normal morphology, rapidly recognized by glial cells, and finally transferred to attenuated processes of microglia and astrocytes [44]. These results suggest that glial processes can recognize the misfolded monomeric or oligomeric membrane proteins accumulated in A*β* amyloidosis which contributes to the cell death and neurotoxicity during AD and prion disease through interaction with cellular prion protein and stress-inducible phosphoprotein-1 [45].

More and more studies suggest that the involvement of glial cells in AD is related with the TRPM2 channel and through inflammation and oxidative stress which are highly involved in the pathology of AD. It is found that TRPM2 channel contributes to the trauma-induced oxidative stress, neuronal apoptosis, mitochondria dysfunction, and [Ca^2+^]_i_ increase [46]; all these changes are related with the pathology of AD. As an antioxidant agent, glutathione is found to play an important role in neuronal oxidant defense and AD [47]. Following aging and during the pathology of AD, glutathione is decreased [47] while the increased current of TRPM2 channel in old culture neurons can be decreased by provision of glutathione [48]. Furthermore, depletion of glutathione can induce oxidative stress, disturbance of Ca^2+^ homeostasis, and apoptosis of hippocampal neurons through activation of TRPM2 channel [49]. The Ca^2+^ influx through TRPM2 channel is linked with the change of glutathione level in microglia and astrocytes [21]. ROS such as H_2_O_2_ can activate TRPM2 channel as plasma membrane channel or intracellular Ca^2+^-release channel [50] to increase intracellular Ca^2+^ and subsequently to induce cell death via poly[ADP-ribose (ADPR)] polymerase (PARP) activation in macrophage cells [51] which are peripheral encounter part of glial cells in the CNS. It is found that ADPR and H_2_O_2_ can elicit a large Ca^2+^ influx, cation current in lipopolysaccharide (LPS) treated microglial cells, and activate the TRPM2 channel expressed in microglia [26]. In a rat stroke model by transient middle cerebral artery occlusion, A*β*, ADPR, and H_2_O_2_ can induce TRPM2 current in microglia [24, 52]. In transcriptional level, oxidative stress and traumatic injury of brain can result in Ca^2+^ influx and enhanced expression of TRPM2 mRNA [27, 53]. Furthermore, oxidative stress induced by inhibition of glutathione biosynthesis can induce human microglia and astrocytes to secrete toxic materials, stimulating them to release TNF-*α*, IL-6, and nitrite ions and to increase the concentration of intracellular Ca^2+^ ([Ca^2+^]_i_) in microglia and astrocytes. These effects are correlated with the activation of inflammatory signaling of p38MAPK, JNK, and NF*κ*B and are reduced by pharmacological blockade of TRPM2 channel or genetic inhibition of TRPM2 channel expression in microglia and astrocytes [21]. These studies suggest that glial TRPM2 channel contributes to AD through inflammation and oxidative stress. Furthermore, recent study indicated that the TRPM2 current in cultured hippocampal neurons can be enhanced by A*β* treatment while TRPM2^−/−^/APP/PS1 transgenic mice demonstrated blockades of increased endoplasmic reticulum stress, age-dependent spatial memory deficit, and reduction of microglial activation although TRPM2^−/−^/APP/PS1 transgenic mice did not show significant change in plaque [20]. These results suggest that deletion of the TRPM2 channel shows protective effect in the AD pathology, which may be achieved through the activation of microglia servicing as the scavenger in the brain and remain to be further studied using specific deletion of TRPM2 channels in glial cells.

## 5. Conclusion

**Figure 1:**
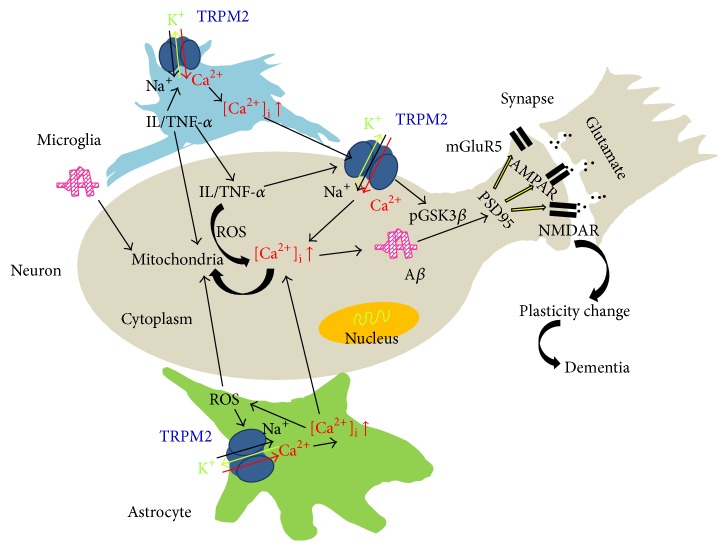
A schematic figure for the involvement of glial TRPM2 channel in plasticity of CNS and AD. We proposed that the activation of TRPM2 channels in microglia and astrocytes produces Ca^2+^ overload and subsequent inflammation and oxidative stress which results in mitochondrial dysfunctions, [Ca^2+^]_i_ increase, A*β* accumulation in neurons, PSD95 reduction, glutamate receptor dysfunction, and finally change of plasticity and dementia. On the other hand, extinct factors such as aging and diabetes can result in increase of extracellular A*β*, which activates the above pathways. The third pathway may be that activation of neuronal TRPM2 channel enhances [Ca^2+^]_i_ and phosphorylates GSK3*β* and subsequent pathway to change plasticity.

As a newly identified nonselective Ca^2+^-permeable channel, the TRPM2 channel is expressed in both neurons and glial cells (mainly microglia and astrocytes). TRPM2 channel can be activated by A*β* and is involved in the synaptic plasticity through interaction with PSD95 and GSK3*β* signal pathway. TRPM2 channel is involved in the plasticity induced by neuropathic and inflammatory pain through glia cells and immune cells. These studies suggest that TRPM2 channel is highly involved in the plasticity of CNS and the pathology of AD through glial cells, as shown in Table 1. According to the schematic figure (Figure 1), we proposed that the activation of TRPM2 channels in microglia and astrocytes produces Ca^2+^ overload and subsequent inflammation and oxidative stress which results in mitochondrial dysfunctions, [Ca^2+^]_i_ increase, A*β* accumulation in neurons, PSD95 reduction, glutamate receptor dysfunction, and finally change of plasticity and dementia. On the other hand, distinct factors such as aging and diabetes can result in increase of extracellular A*β*, which activates the above pathways. The third pathway may be that activation of neuronal TRPM2 channel enhances [Ca^2+^]_i_ and phosphorylates GSK3*β* and subsequent pathway to change plasticity. However, there are still many further studies remaining to be performed to elucidate the detailed mechanism of glial TRPM2 channel in the plasticity of CNS and the pathology of neurodegenerative diseases such as AD, particularly using specific deletion of TRPM2 channel in glial cells. Following the elucidation of the features of the TRPM2 channel in glial cells, it will shed a light on the study of neurodegenerative diseases.

## Figures and Tables

**Table 1:** Major references studying TRPM2 channel in plasticity and AD.

Experimental approach	Effects	Reference
TRPM2 KO hippocampal slice	Deficit in LTD, GSK3*β* inactivation	[39]
TRPM2 KO glia and neuron culture	Glutathione homeostasis loss, inflammation	[21, 48]
TRPM2 KO animal stroke	Neuroprotection, GSK3*β* inhibition	[54]
Expression of TRPM2 in striatal culture, A*β*/oxidative stress	Cell death	[52]
Human microglia culture, rat brain ischemia, inflammation/oxidative stress/electrophysiology	TRPM2 activated in microglia by ADPR	[24]
TRPM2 KO, ROS and inflammation in whole animal	Negative feedback	[19]
Neuropathic and inflammatory pain in TRPM2 KO animal	Inhibition of microglia and pain in KO mice	[17, 22]
Expression of TRPM2 in human glioblastoma, oxidative stress	Promoting cell death	[55]
Electrophysiology in microglia, ADPR/H_2_O_2_	Induction of Ca^2+^ influx and TRPM2 current	[26, 56]
Diabetic rat, brain and DRG	TRPM2 activity and oxidative stress enhanced	[50]
Pharmacological gene deletion of TRPM2 in microglia	TRPM2 mediates inflammation through p38MAPK/JNK	[28]
TRPM2/APP/PS1 KO mice	Absent microglia activation and memory impairment	[20]

## Highlights

TRPM2 channel is expressed in both neurons and glial cells. Glial TRPM2 channel is involved in plasticity in CNS. Glial TRPM2 channel is involved in Alzheimer's disease.

## References

[1] Agostinho P., Cunha R. A., Oliveira C. (2010). Neuroinflammation, oxidative stress and the pathogenesis of Alzheimer's disease. *Current Pharmaceutical Design*.

[2] Chung W. S., Welsh C. A., Barres B. A., Stevens B. (2015). Do glia drive synaptic and cognitive impairment in disease?. *Nature Neuroscience*.

[3] Yürekli V. A., Gürler S., Naziroğlu M., Uğuz A. C., Koyuncuoğlu H. R. (2013). Zonisamide attenuates MPP(+)-induced oxidative toxicity through modulation of Ca^2+^ signaling and caspase-3 activity in neuronal PC12 cells. *Cellular and Molecular Neurobiology*.

[4] Nazıroğlu M. (2012). Molecular role of catalase on oxidative stress-induced Ca^2+^ signaling and TRP cation channel activation in nervous system. *Journal of Receptors and Signal Transduction*.

[5] Latremoliere A., Woolf C. J. (2009). Central sensitization: a generator of pain hypersensitivity by central neural plasticity. *Journal of Pain*.

[6] Chiang C.-Y., Wang J., Xie Y.-F. (2007). Astroglial glutamate-glutamine shuttle is involved in central sensitization of nociceptive neurons in rat medullary dorsal horn. *The Journal of Neuroscience*.

[7] Xie Y. F., Zhang S., Chiang C. Y., Hu J. W., Dostrovsky J. O., Sessle B. J. (2007). Involvement of glia in central sensitization in trigeminal subnucleus caudalis (medullary dorsal horn). *Brain, Behavior, and Immunity*.

[8] Xie Y.-F. (2008). Glial involvement in trigeminal central sensitization. *Acta Pharmacologica Sinica*.

[9] Benfenati F. (2007). Synaptic plasticity and the neurobiology of learning and memory. *Acta Biomedica*.

[10] Welberg L. (2014). Synaptic plasticity: a synaptic role for microglia. *Nature Reviews Neuroscience*.

[11] Yates D. (2014). Synaptic plasticity: microglial cell-mediated depression. *Nature Reviews Neuroscience*.

[12] Bernardinelli Y., Muller D., Nikonenko I. (2014). Astrocyte-synapse structural plasticity. *Neural Plasticity*.

[13] Gonzalez J., Jurado-Coronel J. C., Ávila M. F., Sabogal A., Capani F., Barreto G. E. (2015). NMDARs in neurological diseases: a potential therapeutic target. *International Journal of Neuroscience*.

[14] Kim M. J., Futai K., Jo J., Hayashi Y., Cho K., Sheng M. (2007). Synaptic accumulation of PSD-95 and synaptic function regulated by phosphorylation of serine-295 of PSD-95. *Neuron*.

[15] Hoey S. E., Williams R. J., Perkinton M. S. (2009). Synaptic NMDA receptor activation stimulates *α*-secretase amyloid precursor protein processing and inhibits amyloid-*β* Production. *Journal of Neuroscience*.

[16] Nazıroğlu M. (2011). TRPM2 cation channels, oxidative stress and neurological diseases: where are we now?. *Neurochemical Research*.

[17] Haraguchi K., Kawamoto A., Isami K. (2012). TRPM2 contributes to inflammatory and neuropathic pain through the aggravation of pronociceptive inflammatory responses in mice. *The Journal of Neuroscience*.

[18] Xie Y.-F., MacDonald J. F., Jackson M. F. (2010). TRPM2, calcium and neurodegenerative diseases. *International Journal of Physiology, Pathophysiology and Pharmacology*.

[19] Di A., Gao X. P., Qian F. (2012). The redox-sensitive cation channel TRPM2 modulates phagocyte ROS production and inflammation. *Nature Immunology*.

[20] Ostapchenko V. G., Chen M., Guzman M. S. (2015). The Transient Receptor Potential Melastatin 2 (TRPM2) channel contributes to *β*-amyloid oligomer-related neurotoxicity and memory impairment. *The Journal of Neuroscience*.

[21] Lee M., Cho T., Jantaratnotai N., Wang Y. T., McGeer E., McGeer P. L. (2010). Depletion of GSH in glial cells induces neurotoxicity: relevance to aging and degenerative neurological diseases. *FASEB Journal*.

[22] So K., Haraguchi K., Asakura K. (2015). Involvement of TRPM2 in a wide range of inflammatory and neuropathic pain mouse models. *Journal of Pharmacological Sciences*.

[23] Isami K., Haraguchi K., So K. (2013). Involvement of TRPM2 in peripheral nerve injury-induced infiltration of peripheral immune cells into the spinal cord in mouse neuropathic pain model. *PLoS ONE*.

[24] Fonfria E., Mattei C., Hill K. (2006). TRPM2 is elevated in the tMCAO stroke model, transcriptionally regulated, and functionally expressed in C13 microglia. *Journal of Receptors and Signal Transduction*.

[25] Ohana L., Newell E. W., Stanley E. F., Schlichter L. C. (2009). The Ca^2+^ release-activated Ca^2+^ current (I_CRAC_) mediates store-operated Ca^2+^ entry in rat microglia. *Channels*.

[26] Kraft R., Grimm C., Grosse K. (2004). Hydrogen peroxide and ADP-ribose induce TRPM2-mediated calcium influx and cation currents in microglia. *The American Journal of Physiology—Cell Physiology*.

[27] Bond C. E., Greenfield S. A. (2007). Multiple cascade effects of oxidative stress on astroglia. *Glia*.

[28] Miyake T., Shirakawa H., Kusano A. (2014). TRPM2 contributes to LPS/IFN*γ*-induced production of nitric oxide via the p38/JNK pathway in microglia. *Biochemical and Biophysical Research Communications*.

[29] Chiang C. Y., Zhang S., Xie Y. F. (2005). Endogenous ATP involvement in mustard-oil-induced central sensitization in trigeminal subnucleus caudalis (medullary dorsal horn). *Journal of Neurophysiology*.

[30] Chen X., Lin R., Chang L. (2013). Enhancement of long-term depression by soluble amyloid beta protein in rat hippocampus is mediated by metabotropic glutamate receptor and involves activation of p38MAPK, STEP and caspase-3. *Neuroscience*.

[31] Yashiro K., Philpot B. D. (2008). Regulation of NMDA receptor subunit expression and its implications for LTD, LTP, and metaplasticity. *Neuropharmacology*.

[32] Yamazaki Y., Fujii S. (2015). Extracellular ATP modulates synaptic plasticity induced by activation of metabotropic glutamate receptors in the hippocampus. *Biomedical Research*.

[33] Liu T., Berta T., Xu Z.-Z. (2012). TLR3 deficiency impairs spinal cord synaptic transmission, central sensitization, and pruritus in mice. *Journal of Clinical Investigation*.

[34] Lalo U., Rasooli-Nejad S., Pankratov Y. (2014). Exocytosis of gliotransmitters from cortical astrocytes: implications for synaptic plasticity and aging. *Biochemical Society Transactions*.

[35] Rasooli-Nejad S., Palygin O., Lalo U., Pankratov Y. (2014). Cannabinoid receptors contribute to astroglial Ca^2+^-signalling and control of synaptic plasticity in the neocortex. *Philosophical Transactions of the Royal Society B: Biological Sciences*.

[36] Hayashi Y., Nakanishi H. (2013). Synaptic plasticity and synaptic reorganization regulated by microglia. *Nihon Shinkei Seishin Yakurigaku Zasshi*.

[37] Parkhurst C. N., Yang G., Ninan I. (2013). Microglia promote learning-dependent synapse formation through brain-derived neurotrophic factor. *Cell*.

[38] Koga K., Descalzi G., Chen T. (2015). Coexistence of two forms of LTP in ACC provides a synaptic mechanism for the interactions between anxiety and chronic pain. *Neuron*.

[39] Xie Y.-F., Belrose J. C., Lei G. (2011). Dependence of NMDA/GSK-3*β* mediated metaplasticity on TRPM2 channels at hippocampal CA3-CA1 synapses. *Molecular Brain*.

[40] Azevedo E. P., Ledo J. H., Barbosa G. (2013). Activated microglia mediate synapse loss and short-term memory deficits in a mouse model of transthyretin-related oculoleptomeningeal amyloidosis. *Cell Death and Disease*.

[41] Frozza R. L., Bernardi A., Hoppe J. B. (2013). Neuroprotective effects of resveratrol against A*β* administration in rats are improved by lipid-core nanocapsules. *Molecular Neurobiology*.

[42] Jo S., Yarishkin O., Hwang Y. J. (2014). GABA from reactive astrocytes impairs memory in mouse models of Alzheimer's disease. *Nature Medicine*.

[43] Ko C.-Y., Wang W.-L., Wang S.-M., Chu Y.-Y., Chang W.-C., Wang J.-M. (2014). Glycogen synthase kinase-3*β*-mediated CCAAT/enhancer-binding protein delta phosphorylation in astrocytes promot es migration and activation of microglia/macrophages. *Neurobiology of Aging*.

[44] Jeffrey M., McGovern G., Barron R., Baumann F. (2015). Membrane pathology and microglial activation of mice expressing membrane anchored or membrane released forms of A*β* and mutated human Alzheimer's precursor protein (APP). *Neuropathology and Applied Neurobiology*.

[45] Ostapchenko V. G., Beraldo F. H., Mohammad A. H. (2013). The prion protein ligand, stress-inducible phosphoprotein 1, regulates amyloid-beta oligomer toxicity. *Journal of Neuroscience*.

[46] Yürüker V., Nazıroğlu M., Şenol N. (2014). Reduction in traumatic brain injury-induced oxidative stress, apoptosis, and calcium entry in rat hippocampus by melatonin: possible involvement of TRPM2 channels. *Metabolic Brain Disease*.

[47] Saharan S., Mandal P. K. (2014). The emerging role of glutathione in Alzheimer's disease. *Journal of Alzheimer's Disease*.

[48] Belrose J. C., Xie Y.-F., Gierszewski L. J., MacDonald J. F., Jackson M. F. (2012). Loss of glutathione homeostasis associated with neuronal senescence facilitates TRPM2 channel activation in cultured hippocampal pyramidal neurons. *Molecular Brain*.

[49] Övey I. S., Naziroğlu M. (2015). Homocysteine and cytosolic GSH depletion induce apoptosis and oxidative toxicity through cytosolic calcium overload in the hippocampus of aged mice: involvement of TRPM2 and TRPV1 channels. *Neuroscience*.

[50] Sözbir E., Nazıroğlu M. (2015). Diabetes enhances oxidative stress-induced TRPM2 channel activity and its control by N-acetylcysteine in rat dorsal root ganglion and brain. *Metabolic Brain Disease*.

[51] Zou J., Ainscough J. F., Yang W. (2013). A differential role of macrophage TRPM2 channels in Ca^2+^ signaling and cell death in early responses to H_2_O_2_. *American Journal of Physiology—Cell Physiology*.

[52] Fonfria E., Marshall I. C. B., Boyfield I. (2005). Amyloid *β*-peptide(1-42) and hydrogen peroxide-induced toxicity are mediated by TRPM2 in rat primary striatal cultures. *Journal of Neurochemistry*.

[53] Cook N. L., Vink R., Helps S. C., Manavis J., van den Heuvel C. (2010). Transient receptor potential melastatin 2 expression is increased following experimental traumatic brain injury in rats. *Journal of Molecular Neuroscience*.

[54] Alim I., Teves L., Li R., Mori Y., Tymianski M. (2013). Modulation of NMDAR subunit expression by TRPM2 channels regulates neuronal vulnerability to ischemic cell death. *The Journal of Neuroscience*.

[55] Ishii M., Oyama A., Hagiwara T. (2007). Facilitation of H_2_O_2_-induced A172 human glioblastoma cell death by insertion of oxidative stress-sensitive TRPM2 channels. *Anticancer Research*.

[56] Hecquet C. M., Malik A. B. (2009). Role of H_2_O_2_-activated TRPM2 calcium channel in oxidant-induced endothelial injury. *Thrombosis and Haemostasis*.

